# Microencapsulation of roselle (*Hibiscus sabdariffa* L.) anthocyanins: Effects of drying conditions on some physicochemical properties and antioxidant activities of spray‐dried powder

**DOI:** 10.1002/fsn3.2659

**Published:** 2021-12-08

**Authors:** Quoc‐Duy Nguyen, Thanh‐Thuy Dang, Thi‐Van‐Linh Nguyen, Thi‐Thuy‐Dung Nguyen, Nhu‐Ngoc Nguyen

**Affiliations:** ^1^ Faculty of Environmental and Food Engineering Nguyen Tat Thanh University Ho Chi Minh City Vietnam

**Keywords:** antioxidant activities, encapsulation efficiency, phenolic content, Roselle anthocyanins, spray drying

## Abstract

Anthocyanins are important phytochemical compounds in nature that are of interest not only for their health benefits such as antioxidant, anti‐inflammatory, and anti‐carcinogenic properties, but also for their role in imparting attractive and characteristic color to food products. In this study, anthocyanins from hibiscus (*Hibiscus sabdariffa* L.) calyces were microencapsulated by spray‐drying technique using maltodextrin as the carrier. The experiment was carried out in the full factorial design with two factors, namely inlet temperature (150, 160, and 170°C) and anthocyanin to maltodextrin mass ratio (1:50, 1:60, 1:70, 1:80, 1:90, and 1:100) with the aim of investigating the effect of spray drying conditions on phenolic content, anthocyanin, antioxidant activity, and color of spray‐dried hibiscus powder. The results showed that increasing the carrier ratio significantly reduced the antioxidant content and their activities in the powder. However, the high level of carriers exhibited a protective effect in encapsulating anthocyanin compounds into the maltodextrin matrix, which was demonstrated by high encapsulation efficiency (>85%) observed in the samples prepared at a ratio of 1:100. It should be highlighted that although high temperature (170°C) reduced total anthocyanin concentration, it actually enhanced total phenolic content. In addition, the moisture content of the powder declined with increasing carrier ratio and inlet temperature, and it was found to be in the range of 5.57%–10.19% in the powder. With solubility greater than 93.71%, the total phenolic and total anthocyanin content of spray‐dried hibiscus powder were 31.5–41.9 (mg gallic acid equivalent/g of dry powder) and 6.08–10.47 (mg cyanidin‐3‐glucoside/g of dry powder), respectively.

## Graphical abstract

Increasing the carrier ratio reduced antioxidant contents and activities. High inlet temperature effectively retained phenolic compounds. High encapsulation efficiency of anthocyanin (above 85%) was obtained at an ACN:MD ratio of 1:100 (w/w). Increasing carrier ratio and inlet temperature resulted in decrease in moisture content, and the solubility of all samples exceeded 93.71%.

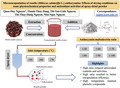

## 1 INTRODUCTION

Anthocyanins, which are members of the flavonoid family, have the ability to absorb light in the ultraviolet and visible regions and are responsible for a variety of colors in plant materials (Costa et al., 2015; Pervaiz et al., 2017). The common food commodities containing anthocyanins are purple grapes, cherries, plums, raspberries, strawberries, blackberries, blueberries, black currants, cranberries, chokeberry, red cabbage, and red wine (Wu et al., 2006). Anthocyanins, glycosides of anthocyanidins (also called aglycones) and sugars, are the result of anthocyanidin transformation by attaching glycosyl and aromatic or aliphatic acyl residues to anthocyanidins molecules (Ekici et al., 2014). There are about 17 types of anthocyanidins found in nature, and over 90% of anthocyanins are based on only six types of anthocyanidins, namely pelargonidin, cyanidin, peonidin, delphinidin, petunidin, and malvidin (Espín et al., 2000).

Hibiscus (*Hibiscus sabdariffa* L.), also known locally as roselle, are rich in anthocyanins and can be used as a good source to produce a vibrant red colorant for many foods (Frimpong et al., 2014; Jabeur et al., 2017). The main components in hibiscus flowers are organic acids, anthocyanins, polysaccharides, and flavonoids groups (Da‐Costa‐Rocha et al., 2014; Formagio et al., 2015). In particular, some studies have found that the main components in the anthocyanin group including delphinidin‐3‐sambubioside and cyanidin‐3‐sambubioside exist in hibiscus calyxes and leaf extracts (Borrás‐Linares et al., 2015; Jabeur et al., 2017). Besides, anthocyanin is the biological compound of most interest because of its health benefits, antioxidant, and anti‐cancer potential (Zhang et al., 2014). As antioxidants, anthocyanins are chemically unstable compounds (Jung et al., 2020). The stability of anthocyanins is affected by factors such as temperature, light, oxygen content, pH, chemical structure, solvents, enzymes, and metal ions (Castañeda‐Ovando et al., 2009; Ekici et al., 2014). Due to their low stability under environmental conditions during processing and storage, the introduction of such compounds into foods is challenging (Mahdavi et al., 2014). Therefore, the microencapsulation process is a promising method to overcome the stability limitation of anthocyanin pigments (Lu et al., 2015).

Microencapsulation is a popular technique used in the pharmaceutical industry, which has been used to protect solid, liquid, or gaseous core compounds from adverse environmental conditions, such as light, moisture, and oxygen, thereby contributing to an increase in the shelf life of the product and promoting a controlled release of the encapsulate (Pourashouri et al., 2014). Microencapsulation methods are chosen based on specific applications and factors such as particle size, physicochemical characteristics of core and wall materials, release mechanisms, and process cost (Murugesan & Orsat, 2012). Most microcapsules are small spheres with diameters of between 1 and 1,000 μm (Mahdavi et al., 2014). Among the various techniques used for microencapsulation, spray drying is one of the most widely used since it provides rapid evaporation of water and maintains the low temperature in the particles (Fang & Bhandari, 2012). During operation, the spray drying conditions, such as inlet temperature, the feed flow rate, pressure of atomizing air, material viscosity, type of wall material, under which they are conducted, greatly affect the effectiveness of the drying process and the final product properties (Moser et al., 2017; Wang et al., 2015). Spray drying is the most common technique used to encapsulate anthocyanins (Mahdavi et al., 2014).

Maltodextrin is a hydrolyzed starch produced by partial hydrolysis of starch with acid or enzyme commonly used as a raw material in the process of microencapsulation of food ingredients (Gharsallaoui et al., 2007; Goula & Adamopoulos, 2012). Maltodextrin is considered as a good microencapsulation agent because it exhibits a low viscosity at high solid content and good solubility (Moser et al., 2017). Maltodextrin is mainly used as additives during drying of fruit juice, increasing the glass transition temperature, reducing the stickiness, and enhancing stability of the powder (Apintanapong & Noomhorm, 2003).

In this study, the effects of inlet temperature and ratio of anthocyanins to maltodextrin on phenolics, anthocyanins, antioxidant activity, and some physical characteristics of spray‐dried hibiscus powder were investigated. Encapsulation efficiency was also measured and compared across samples with the goal of microencapsulating anthocyanins within maltodextrin powder. In addition, Pearson correlation was also used to evaluate the relationship between these responses.

## 2 MATERIALS AND METHODS

### 2.1 Materials and chemicals

Dried hibiscus calyces were purchased from Viet Hibiscus Company (Ho Chi Minh City, Vietnam). The plants were grown in Bien Hoa City (Dong Nai Province, Vietnam). After harvesting, fresh flowers were air‐dehydrated by convection using hot air at 60°C. Dried products were stored in polyethylene bags in a cool, dry place, away from direct sunlight.

Gallic acid, 2,2‐diphenyl‐1‐picrylhydrazyl (DPPH), 2,4,6‐tripyridyl‐s‐triazine (TPTZ), 2,2′‐azino‐bis(3‐ethylbenzothiazoline‐6‐sulphonic acid) (ABTS), 2,9‐dimethyl‐1,10‐phenanthroline (neocuproine), and 6‐hydroxy‐2,5,7,8 tetramethylchroman‐2‐carboxylic acid (Trolox) were purchased from Sigma‐Aldrich (Singapore). Folin–Ciocalteu reagent (2 N) was prepared basically from solid sodium tungstate, sodium molybdate, and lithium sulfate.

Maltodextrin (DE 8–10, solubility >98%) was purchased from Baolingbao Biology Co., Ltd. (Shandong, China). Sodium acetate trihydrate, acetic acid, sodium carbonate, methanol, ethanol, potassium persulfate, phosphoric acid, hydrochloric acid, potassium chloride, ferric chloride hexahydrate, cuprous chloride, ammonium acetate, and other chemicals were of analytical grade.

### 2.2 Preparation of roselle anthocyanin extracts

250 g of dried roselle calyces were extracted at the temperature of 50°C for 30 min in 400 ml of 70% (v/v) ethanol acidified to pH 2.0 by 2 N hydrochloric acid using the HI 2211–02 pH meter (Hanna Instruments, Mauritius, Romania). After extraction, the extract was obtained by filtering through Whatman No. 2 filter paper. The filtrate was then concentrated under vacuum conditions in the Hei‐VAP Value rotary vacuum evaporator (Heidolph Instruments, Schwabach, Germany) at 55°C to remove the ethanol solvent. To determine the amount of carrier required in the microencapsulation process, the concentrate was preliminarily analyzed for total monomeric anthocyanin content. The results showed that the concentrated roselle extract contained anthocyanin with the content of 1.08 g of cyanidin‐3‐glucoside /L.

### 2.3 Spray drying of anthocyanin extracts

The concentrated anthocyanin extract was mixed with maltodextrin according to the mass ratio of anthocyanin (ACN) to maltodextrin (MD) which were at 1:50, 1:60, 1:70, 1:80, 1:90, and 1:100 (w/w). Then, spray drying was conducted in the *SD*‐06AG spray drier (Lab Plant Ltd.). The feed flow rate was set at 500 ml/h, and the inlet temperatures were investigated at three levels of 150°C, 160°C, and 170°C with outlet temperatures of 91°C, 98°C, and 99°C, respectively. After spray drying, the powdered samples were refrigerated at 4°C in polyethylene bags for further analysis.

### 2.4 Analytical methods

#### 2.4.1 Total anthocyanin content (TAC)

The total anthocyanin content (TAC) of spray‐dried powder was measured according to the method described in the literature with slight modification (Mahdavi et al., 2016). Briefly, 100 mg of the sample was weighed and mixed with 1 ml of distilled water. The sample was then crushed with a set of pestle and mortar to destroy the microstructure. 10 ml of 96% ethanol was used to extract for 5 min. The filtrate was collected and analyzed for TAC using total monomeric anthocyanin assay.

Total monomeric anthocyanin content was determined using differential pH method (Pantelidis et al., 2007). Two buffer solutions were used to dilute the sample solution: 0.2 M KCl buffer (pH 1.0) and 0.1 M acetate buffer (pH 4.5). The absorbances of the two samples were determined using the UV‐1800 spectrophotometer (Shimadzu Inc.) at two different wavelengths of 520 and 700 nm. The total monomeric anthocyanin content was calculated using the formula: [(A520 – A700) pH1.0 – (A520 – A700) pH4.5] × *df* ×1000 × 26,900/449.2; where, A is the absorbance of the sample, *df* is the dilution factor, 26,900 is the extinction coefficient, 449.2 is molecular mass of cyanidin‐3 glucoside, and 1000 is the g to mg conversion coefficient. The results were expressed as mg of cyanidin‐3‐glucoside/g of sample on the dry weight (mg C3G/g DW).

#### 2.4.2 Surface anthocyanin content (SAC)

The surface anthocyanin content (SAC) of spray‐dried powder was measured based on the method described in the literature with slight modification (Mahdavi et al., 2016). Briefly, 100 mg of the sample was weighed and mixed with 10 ml of 96% ethanol. After vortexed for 20 s in the VELP ZX4 advanced IR vortex mixer (VELP Scientifica) and centrifuged in the PLC‐05 centrifuge (Gemmy Industrial Corp.) at 1,277 *g* for 3 min, the supernatant was collected and filtered through a membrane filter with pore size of 0.45 μm. The filtrate was collected and analyzed for SAC using total monomeric anthocyanin assay. In addition, encapsulation efficiency (%) was calculated as (TAC – SAC) × 100/TAC.

#### 2.4.3 Total phenolic content (TPC)

The phenolic content (TPC) was performed according to the Folin–Ciocalteu method described in ISO 14502–1:2005 (ISO, 2005). Briefly, 100 mg of the sample was weighed and mixed with 10 ml of distilled water. To determine the phenolic content, 0.6 ml of the diluted sample was added to 1.5 ml of Folin–Ciocalteu reagent (10‐fold dilution) and incubated in the dark for 5 min. After adding 1.2 ml of 7.5% (w/v) Na_2_CO_3_, the reaction mixture was incubated for 60 min in the dark. The absorbance was then measured at 765 nm using the UV‐Vis spectrophotometer, and the distilled water was used as blank. Total phenolic content was calculated using the gallic acid calibration curve and expressed as mg gallic acid equivalent/g of sample on the dry weight (mg GAE/g DW).

#### 2.4.4 DPPH free radical scavenging activity

DPPH free radical scavenging assay was performed according to the method described in the literature with some modifications (Brand‐Williams et al., 1995). Briefly, 100 mg of the sample was weighed and mixed with 10 ml of distilled water. To prepare the stock DPPH reagent, 0.024 g of DPPH was dissolved in 100 ml of methanol and stored at 4°C for 24 hr. The working reagent was then prepared by mixing the stock DPPH solution with methanol so that the absorbance of the mixture reaches 1.10 ± 0.02 at 515 nm with the UV‐Vis spectrophotometer. To determine the DPPH free radical reduction activity, 2.85 ml of the working DPPH reagent was added to 0.15 ml of the diluted sample. The reaction mixture was incubated for 30 min in the dark, and the absorbance was measured at 515 nm over methanol blank. The percentage of DPPH free radical inhibition was calculated using the following formula: % Inhibition = (1 – Absorbance of sample/Absorbance of control) × 100. DPPH antioxidant activity was calculated against the Trolox concentration–% inhibition curve and expressed as mg Trolox equivalent/g of sample on the dry weight (mg TE/g DW).

#### 2.4.5 ABTS^•+^ cation radical scavenging ability

ABTS^•+^ cation radical scavenging assay was performed according to the method described in the literature with some modifications (Arnao et al., 2001). Briefly, 100 mg of the sample was weighed and mixed with 10 ml of distilled water. To prepare the stock ABTS^•+^ reagent, 7.4 mM ABTS solution was mixed with 2.6 mM potassium persulfate solution at a ratio of 1:1 (v/v) and then refrigerated at 4°C for 24 h. The working reagent was prepared by mixing the stock ABTS^•+^ solution with methanol so that the absorbance of the mixture reaches 1.10 ± 0.02 at a wavelength of 734 nm using the UV‐Vis spectrophotometer. To determine the ABTS^•+^ cation radical scavenging activity, 2.85 ml of the stock ABTS^•+^ reagent was added to 0.15 ml of the diluted sample. The reaction mixture was incubated for 30 min in the dark and the absorbance was measured at 734 nm using the UV‐Vis spectrophotometer over methanol blank. The percentage of ABTS^•+^ cation radical inhibition was calculated using the formula: % Inhibition = (1 – Absorbance of sample/Absorbance of control) × 100. The ABTS^•+^ cation radical scavenging ability was calculated against the Trolox concentration–% inhibition curve and expressed as mg Trolox equivalent /g of sample on the dry weight (mg TE/g DW).

#### 2.4.6 Ferric reducing antioxidant power (FRAP)

The ferric reducing antioxidant power (FRAP) assay was performed according to the method described in the literature with some modifications (Benzie & Strain, 1996). Briefly, 100 mg of the sample was weighed and mixed with 10 ml of distilled water. To prepare the reagent, the acetate buffer (0.3 M, pH 3.6), TPTZ (10 mM in 40 mM HCl), and FeCl_3_.6H_2_O (20 mM) solution were mixed at a 10:1:1 (v/v) ratio. To determine the ferric reducing antioxidant power (FRAP), 2.85 ml of FRAP reagent was added to 0.15 ml of diluted sample. The reaction mixture was incubated for 30 min in the dark, and the absorbance was measured at 593 nm with the UV‐Vis spectrophotometer over distilled water as blank. The ferric reducing antioxidant power (FRAP) was calculated based on the Trolox calibration curve and expressed as mg Trolox equivalent/g of sample on the dry weight (mg TE/g DW).

#### 2.4.7 Cupric reducing antioxidant capacity (CUPRAC)

Cupric reducing antioxidant capacity (CUPRAC) assay was performed according to the method described in the literature with some modifications (Apak et al., 2006). Briefly, 100 mg of the sample was weighed and mixed with 10 ml of distilled water. Sample solution (0.5 ml) was added to premixed reaction mixture containing CuCl_2_ (1 ml, 10 mM), neocuproine (1 ml, 7.5 mM), and ammonium acetate buffer (1 ml, 1 M, pH 7.0) adjusted to pH 7.0. The reaction mixture was incubated for 30 min in the dark, and the absorbance was measured at 450 nm with the UV‐Vis spectrophotometer over distilled water as blank. The cupric reducing antioxidant capacity (CUPRAC) was calculated based on the Trolox calibration curve and expressed as mg Trolox equivalent/g of sample on the dry weight (mg TE/g DW).

#### 2.4.8 Moisture content

The moisture content was determined based on the method described in literature (de Almeida Paula et al., 2019). The beads (2 g) were weighed and spread evenly on the petri dishes. The sample was then dried at 105°C until reaching constant mass in the LO‐FS100 forced convection oven (LK Lab, Namyangju, Korea) to constant mass.

#### 2.4.9 Solubility

The solubility of powder was determined based on the method described in literature with slight changes (Cano‐Chauca et al., 2005). Briefly, 0.1 g sample was mixed with 10 ml of distilled water, and the mixture was stirred in the magnetic stirrer for 5 min. Then, the solution was centrifuged at 3000 rpm for 10 min. The supernatant was transferred to a petri dish and dried at 105°C until it reached constant mass. Solubility was calculated as the difference in solid weight between the reconstituted solution and the original solid sample.

#### 2.4.10 Color attributes

The values of L*, a*, and b* were measured by a CR‐400 colorimeter (Konica Minolta Sensing, Inc.) using the Hunter CIELAB color system (Pérez‐Caballero et al., 2003). Values of L*, a*, and b* are measured to describe a three‐dimensional color space in which the vertical axis L* is a measure of brightness, with values ranging from completely opaque (0) to fully transparent (100); (+/–) a* is a measure of redness and greenness, and (+/–) b* is a measure of yellowness and blueness. These values were then used to calculate chroma (C*) and hue angle (hº) values, according to the following formula:

(1) $$\text{Chroma}\;\left(C*\right)=\text{sqrt}\;\left(a^{*\wedge 2}+b^{*\wedge 2}\right)$$

(2) $$\text{Hue}\left(h^{\circ }\right)=\arctan\left(\frac{\text{b*}}{\text{a*}}\right)$$

The hue of a color is measured as an angle around the circle, with red being at 0º, orange‐yellow at 90º, green at 180º, and blue at 270º. In addition, chroma provides more information about the saturation or intensity of the color (Wojdyło et al., 2019).

### 2.5 Statistical analysis

Experimental data were analyzed using SPSS 15 software (SPSS Inc.) using basic statistical techniques. Pearson correlation and analysis of variance were used to determine the differences between samples, and Tukey's Multiple Range test was applied to determine significant differences between mean values at the significance level of 5%. All experiments were conducted in triplicate.

## 3 RESULTS AND DISCUSSION

### 3.1 Effects of inlet temperature and ACN:MD ratio on TPC

**FIGURE 1:**
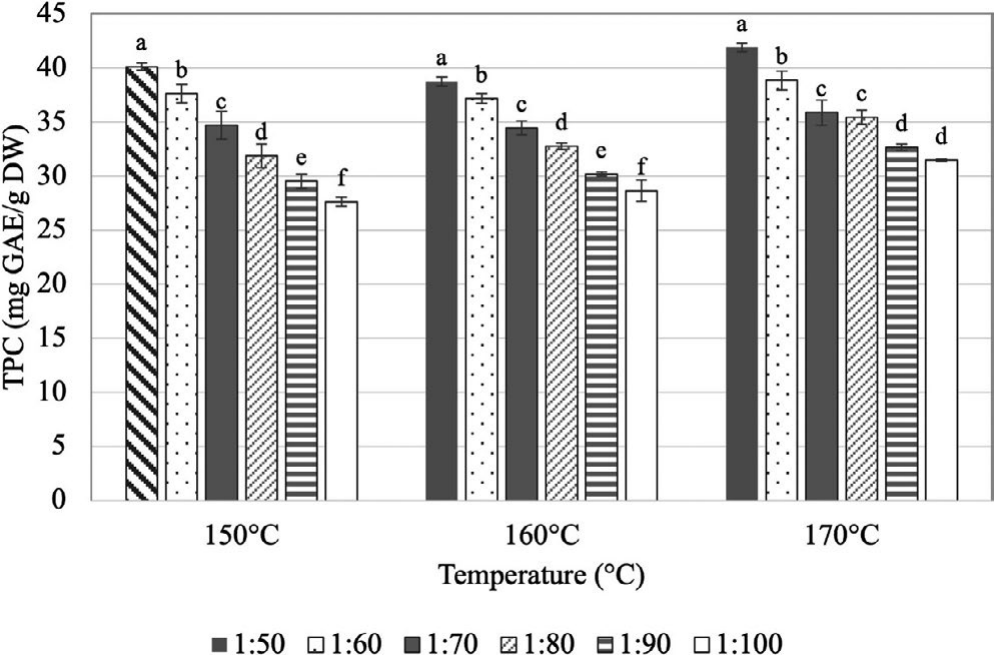
Effects of inlet temperature (°C) and ratio (w/w) of anthocyanin (ACN) to maltodextrin (MD) on total phenolic content (TPC, mg GAE/g DW) of spray‐dried roselle powder. Note: Different letters within each method indicate that the mean values were significantly different at 95% confidence level

Along with the well‐known anthocyanins, hibiscus calyces contain a variety of other phenolic compounds in high concentrations, including hibiscus acid derivatives, hydroxybenzoic acids, caffeoylquinic acids, and flavonols (Ramírez‐Rodrigues et al., 2012). As a result, it is essential to evaluate the impact of spray‐drying conditions on the antioxidant content and activity of hibiscus powder in order to maintain the health benefits associated with spray‐dried hibiscus powder. The effect of spray drying conditions on the TPC of spray‐dried roselle powder is shown in Figure 1. The results exhibited that when the quantity of maltodextrin in the feed solution was increased, the TPC of hibiscus spray‐dried powder significantly dropped. Specifically, at two examined temperatures of 150°C and 160°C, raising the ratio of ACN:MD from 1:50 to 1:100 (w/w) resulted in a 30% reduction in TPC. It is noteworthy that the phenolic content (31.5–41.9 mg GAE/g DW) was better preserved at the higher inlet temperature 170°C than at the lower inlet temperature. Furthermore, increasing the carrier ratio from 1:90 to 1:100 had no effect on the phenolic concentration at this temperature.

It is obvious that the antioxidant content of hibiscus extract, such as phenolics or anthocyanins, decreased with increasing carrier concentration in the hibiscus extract prior to drying, as demonstrated by dilution of these compounds with the maltodextrin. Caliskan and Dirim (2016) concluded that increasing the inlet temperature in the range of 160–180°C reduced the TPC while the temperature range of 180–200°C increased this value by 16% during the spray drying of sumac extracts. Similar findings were observed in the study conducted by Mishra et al. (2014) in which encapsulation of phenolic extracts from amla (*Emblica officinalis*) using maltodextrin (DE 10) at spray drying temperature from 125°C to 200ºC and the carrier ratio from 5% to 9% was conducted. The study revealed that when the spray‐drying temperature was raised, the TPC dropped substantially. In detail, TPC declined as temperature rose from 125°C to 175°C; however, TPC increased when temperature rose from 175°C to 200°C. The increase in TPC at temperatures above 175°C is possibly due to polymerization of phenolic compounds of the powder (Mishra et al., 2014).

### 3.2 Effects of inlet temperature and ACN:MD ratio on TAC, SAC, and EE

**FIGURE 2:**
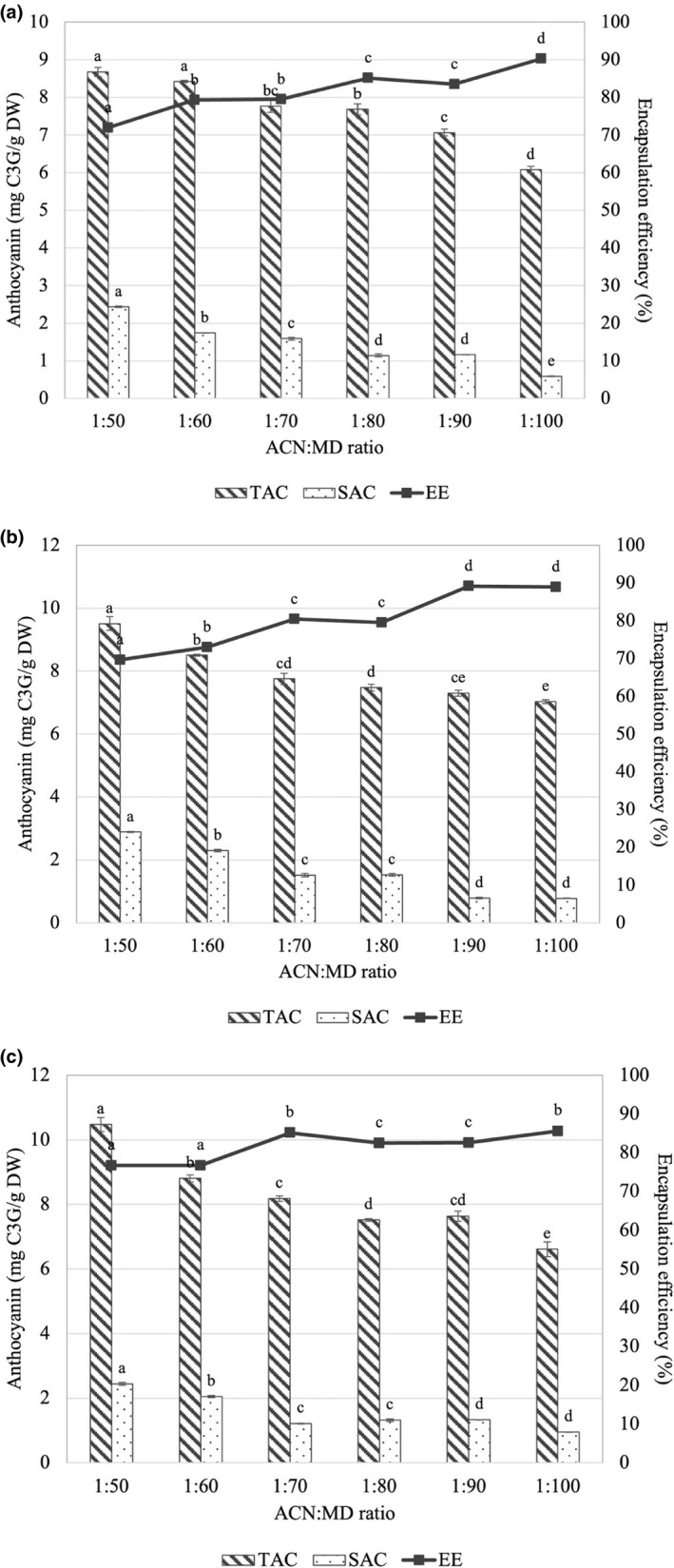
Effects of inlet temperature (°C) and ratio (w/w) of anthocyanin (ACN) to maltodextrin (MD) on total anthocyanin (TAC, mg C3G/g DW), surface anthocyanin (SAC, mg C3G/g DW) contents and encapsulation efficiency (EE, %) of spray‐dried roselle powder at (a) 150°C, (b) 160°C, and (c) 170°C. Note: Different letters within each method indicate that the mean values were significantly different at 95% confidence level

Effects of carrier ratio and spray drying temperature on TAC and SAC of spray‐dried roselle powder are exhibited in Figure 2. The results showed that at low carrier ratio values (from 1:50 to 1:70), the TAC value altered in a comparable way to the total phenolic content, indicating that the high temperature maintained the anthocyanin content better. TAC, on the other hand, diminished with increasing drying temperature at high carrier concentration levels. In all samples, the SAC value is very low when compared to the TAC value. SAC is a quantity of anthocyanin that is not located inside the microcapsule wall and remains on the microcapsule surface. As a result, lower SAC indicates better microcapsule characteristics (Laokuldilok & Kanha, 2017). This is the reason leading to the result that EE was higher than 70% in all samples. In addition, it is important to note that increasing the quantity of carrier has a beneficial impact on the EE and high drying temperatures will help reduce the amount of maltodextrin required to entrap ACN within the matrix of the spray‐dried powder.

Increasing the inlet temperature leads to an increase in anthocyanin loss due to the thermal sensitivity of this compound. Powders dried at low inlet temperatures tended to agglomerate due to their low moisture content, reducing the exposure of active compounds to oxygen and protecting them from oxidation (Quek et al., 2007; Tonon et al., 2008). According to Do and Nguyen (2018), the evaporation process has been expedited by raising the inlet air temperature; a more stable wall has been produced, which acts as a matrix that protects the anthocyanin core from heat treatment. The high inlet air temperature leads to the rapid formation of semi‐permeable layers on the surface of water droplets, helping to retain the core material (Kalamara et al., 2015). However, inlet air temperatures above 180ºC cause excessive bubble formation associated with imperfect surface structures, which increases losses during spray drying (Mohammed et al., 2017). TAC, on the other hand, has been shown to drop substantially when temperatures increased too high (Do & Nguyen, 2018). Spray‐drying conditions carried out at high temperatures may lead to a significant loss of red pigments due to decomposition and formation of undesirable brown or colorless compounds (Jafari et al., 2017).

Encapsulation efficiency is an important indicator to evaluate the efficiency of the microencapsulation process. This value is calculated based on the difference in concentration of the desired active ingredient on the surface compared with the entire particle. In this study, the active compound chosen as the target was anthocyanin. Effects of the ratio of wall materials and spray‐drying temperature on the encapsulation efficiency of anthocyanin from roselle calyces are exhibited in Figure 2. The results showed that the EE of all samples was relatively high and ranges from 69.95% to 90.27%. Furthermore, at higher inlet temperatures, the amount of carrier required for effective microencapsulation was reduced. Specifically, at temperature 160°C, the required carrier ratio was in the range from 1:90 to 1:100 while at 170°C, the required carrier ratio was 1:70 (w/w). High EE values using maltodextrin as carrier have been reported in studies of anthocyanin microencapsulation of plant materials such as roselle (99.69%) (Idham et al., 2012), jabuticaba (83.21%–99.02%) (Silva et al., 2013), and blueberry (74.40%–85.22%) (da Rosa et al., 2019). Differences in EE can be attributed to the nature of wall materials as well as operating conditions including inlet temperature, outlet temperature, and feed flow rate (Tupuna et al., 2018). Upon the microencapsulation by spray drying, the increase in temperature led to a decrease in the protective effect of maltodextrin at the same DE value (Fredes et al., 2018; Laokuldilok & Kanha, 2015). According to Minemoto et al. (2002), at a low carrier ratio, the amount of wall material may not be sufficient to completely cover the droplets of core material and this deficiency can lead to a decrease in encapsulation efficiency.

### 3.3 Effects of inlet temperature and ACN:MD ratio on antioxidant activities

**FIGURE 3:**
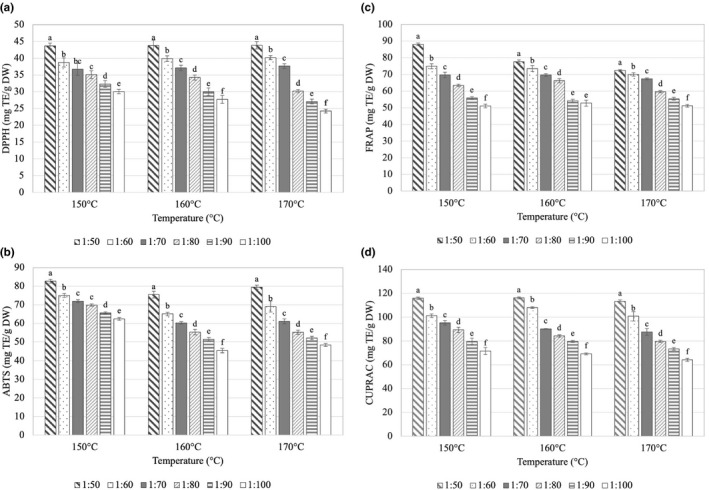
Effects of inlet temperature (°C) and ratio (w/w) of anthocyanin (ACN) to maltodextrin (MD) on antioxidant activities (mg TE/g DW) of spray‐dried roselle powder: (a) DPPH radical scavenging activity, (b) ABTS cation radical reduction ability, (c) ferric reducing antioxidant power (FRAP), and (d) cupric reducing antioxidant capacity (CUPRAC). Note: Different letters within each method indicate that the mean values were significantly different at 95% confidence level

The presence of various antioxidants, each with a distinct mechanism of action, determines antioxidant capacity of food ingredients and products. As a result, the antioxidant capacity of food items should be evaluated using a variety of assays (Moo‐Huchin et al., 2014). In this study, four different methods were used to determine antioxidant capacity of roselle spray‐dried powder. Effects of carrier ratio and spray drying temperature on antioxidant activities of spray‐dried roselle powder are exhibited in Figure 3. The results showed that the inlet temperature and carrier ratio significantly affected all four antioxidant activity values (*p* < .05). The common thread running across the changes in these four activity values was that they all dropped as the carrier ratio increased, implying that these changes in the antioxidant activities followed a similar pattern as TPC. The combined impact of carrier ratio and inlet temperature on each activity, on the other hand, resulted in substantial variations in each activity. It was discovered that in the range of carrier ratio of 1:50–1:70, there were no differences in DPPH free radical scavenging activity of samples dried at the three inlet temperatures. Increased temperature, however, substantially reduced antioxidant activity at higher carrier ratios. Regarding ABTS^•+^ cation radical scavenging activity, this value was consistent between all samples dried at 160°C and 170°C while those dried at 150°C had greater values. At carrier ratios of 1:90 and 1:100, the change in FRAP values tended to be identical to the DPPH free radical scavenging activity, and the CUPRAC value declined with rising inlet temperature at all carrier ratios.

Apparently, the dilution effect caused by the addition of maltodextrin to the hibiscus extract led to a remarkable reduction in antioxidant activity in samples with high carrier concentration. The negligible antioxidant activity of maltodextrin is the explanation for this phenomenon. This result was similar to that of de Souza et al. (2014) obtained when preparing pigment powder from winemaking pomace. Krishnaiah et al. (2012) also found that when increasing inlet temperature and core:wall material ratio, DPPH radical inhibition and phenolic content decreased significantly. High temperatures might adversely affect the structure of phenolic compounds, causing disruption of the structure, leading to the formation of various compounds, thereby reducing antioxidant activity.

### 3.4 Effects of inlet temperature and ACN:MD ratio on moisture content and solubility

Moisture and solubility are two important parameters that represent the quality of instant powder products. Products with low moisture content and high solubility are considered good quality products (Vardin & Yasar, 2012). A high feed flow rate negatively affects the moisture content of the powder, stemming from the short contact time between the atomized feed and the drying agent, leading to inefficient heat transfer and low water evaporation rate (Tonon et al., 2008). In this study, the feed flow rate was fixed at 500 ml/h based on preliminary investigations. Effects of carrier ratio and spray drying temperature on moisture content and solubility of spray‐dried roselle powder are exhibited in Table 1. The results showed that the moisture content of spray‐dried hibiscus powder was significantly different between the samples while the solubility was not different with values in the range of 93.71%–97.85%. In terms of moisture content, increasing the temperature and carrier ratio significantly reduced the moisture content of the powder. It can be seen that at high carrier ratio (1:90 and 1:100) and high spray drying temperature (160°C and 170°C), the moisture content of the powder was the lowest (about 5.6%).

**TABLE 1:** Effects of inlet temperature (°C) and ratio (w/w) of anthocyanin (ACN) to maltodextrin (MD) on some physical properties of spray‐dried roselle powder

		ACN:MD ratio (w/w)					
		1:50	1:60	1:70	1:80	1:90	1:100
L*	150°C	45.07 (0.50)a	45.36 (0.83)a	47.71 (0.23)b	51.17 (0.41)c	53.27 (0.47)d	52.6 (0.67)cd
	160°C	47.04 (0.18)a	50.55 (0.37)b	51.40 (0.79)bc	52.57 (0.45)cd	53.11 (0.34)d	54.77 (0.46)e
	170°C	50.15 (1.2)a	51.46 (1.02)ab	51.84 (0.62)abc	52.32 (0.54)bc	54.37 (0.33)d	53.78 (0.17)cd
a*	150°C	25.79 (0.38)a	26.50 (0.62)ac	27.97 (0.29)bd	27.75 (0.16)bd	27.58 (0.26)bc	28.90 (0.62)d
	160°C	26.82 (0.85)ab	27.96 (0.04)bc	26.23 (0.67)a	28.68 (0.18)c	28.66 (0.18)c	27.20 (0.23)ab
	170°C	24.53 (0.39)a	26.50 (0.40)b	26.30 (0.26)b	27.20 (0.25)bc	27.80 (0.51)c	27.57 (0.12)c
b*	150°C	8.49 (0.12)a	8.34 (0.08)ab	8.33 (0.06)ab	8.19 (0.19)abc	8.10 (0.15)bc	7.89 (0.01)c
	160°C	8.40 (0.09)a	8.38 (0.18)a	8.08 (0.19)ab	7.9 (0.06)b	8.34 (0.04)a	7.86 (0.11)b
	170°C	7.66 (0.30)a	8.20 (0.15)b	8.11 (0.05)ab	8.23 (0.11)b	8.49 (0.20)bc	8.91 (0.06)c
C*	150°C	27.15 (0.4)a	27.78 (0.60)ac	29.19 (0.26)bd	28.93 (0.20)bcd	28.74 (0.30)bc	29.96 (0.59)d
	160°C	28.11 (0.82)ab	29.19 (0.03)bc	27.45 (0.67)a	29.75 (0.17)c	29.85 (0.17)c	28.31 (0.20)ab
	170°C	25.7 (0.46)a	27.74 (0.35)b	27.53 (0.25)b	28.42 (0.26)bc	29.07 (0.48)c	28.97 (0.12)c
h	150°C	18.22 (0.13)a	17.48 (0.38)b	16.59 (0.25)c	16.44 (0.29)c	16.37 (0.14)c	15.27 (0.32)d
	160°C	17.4 (0.5)a	16.69 (0.36)ab	17.11 (0.33)a	15.41 (0.13)c	16.22 (0.13)bc	16.13 (0.33)bc
	170°C	17.34 (0.39)ab	17.21 (0.51)ab	17.14 (0.21)ab	16.83 (0.22)a	16.98 (0.53)ab	17.91 (0.15)b
W (%)	150°C	10.19 (0.26)a	9.8 (0.08)b	9.71 (0.26)cd	8.93 (0.06)d	7.69 (0.23)e	7.66 (0.39)e
	160°C	9.43 (0.11)a	8.22 (0.05)b	8.79 (0.29)ab	6.93 (0.20)c	5.67 (0.11)d	5.71 (0.13)d
	170°C	8.51 (0.35)a	7.36 (0.11)b	7.52 (0.24)b	6.02 (0.07)c	5.69 (0.24)c	5.57 (0.01)c
S (%)	150°C	96.52 (0.89)a	94.29 (2.80)a	96.03 (2.09)a	97.50 (2.17)a	97.41 (1.29)a	96.70 (2.69)a
	160°C	97.13 (1.75)a	97.85 (1.54)a	95.92 (0.67)a	96.23 (2.23)a	95.95 (2.29)a	97.22 (1.48)a
	170°C	93.71 (3.1)a	96.05 (2.56)a	97.68 (1.54)a	97.54 (2.57)a	96.83 (2.95)a	94.91 (1.98)a

Note

Results were expressed as mean (deviation), and in the same column, the different letter symbols indicate that the mean values were significantly different (*p* < .05).

Abbreviations: S, solubility (%); W, moisture content (%).

The moisture content of spray‐dried hibiscus powder was similar to other studies on spray drying of blueberry (4.77%–7.00%) (da Rosa et al., 2019), guava (2.14%–2.24%) (Mahendran, 2011), watermelon (1.49%–2.78%) (Quek et al., 2007), pomegranate (2.4%–4.0%) (Vardin & Yasar, 2012), and sumac (1.89%–2.94%) (Caliskan & Dirim, 2016) extracts. Krishnaiah et al. (2012) studied the spray‐drying process of *Morinda citrifolia* L. extract using maltodextrin as a carrier with an inlet temperature in the range of 90–140°C. Research showed that high inlet temperatures increased the accumulation of moist particles on the dryer wall and reduced product recovery. However, the low temperature provided insufficient heat to evaporate the majority of water in the feed, producing the product with high moisture content and easy to stick to the chamber wall. The moisture content of the powder decreased at high inlet temperature which causes an increase in heat transfer rate leading to an increase in moisture removal rate. Low moisture content is also a result of increased carrier content in the feed by reducing the amount of water that needs to be evaporated during the drying process. However, excessively increasing the amount of carrier significantly reduces the quality of the spray‐drying powder as well as increases the feed viscosity, resulting in a decrease in product recovery (Tonon et al., 2008). The phenomenon of sticking to the wall of the powder occurs possibly because the content of simple sugars and organic acids present in the raw materials reduces the glass transition temperature of the mixture. Therefore, the addition of high‐molecular‐weight polymers such as maltodextrin increased the glass transition temperature and overcame the stickiness (Caliskan & Dirim, 2016). In addition, high solubility is an inevitable result when spray‐dried hibiscus powder was produced from maltodextrin with a solubility above 98% and the extract has been filtered to remove insoluble components.

### 3.5 Effects of inlet temperature and ACN:MD ratio on color attributes

Color is one of the important criteria in food products which affects not only the overall quality but also the purchase intention of consumers (Gerard & Roberts, 2004). Effects of carrier ratio and spray‐drying temperature on color parameters of spray‐dried roselle powder are exhibited in Table 1. The results showed that increasing the carrier ratio increased the brightness of spray‐dried hibiscus powder. This is due to the initial white color of maltodextrin when integrated with hibiscus extracts. In addition, the L* value at the three investigated temperatures showed insignificant differences. The a* and b* values were also not significantly different between the samples, ranging from 24.53–28.90 and 7.66–8.91, respectively. Since the differences in a* and b* were minimal, the differences in C* and h° of the samples were inappreciable and the color of hibiscus powder still had a characteristic red color as shown by the h° value in the range 15.27–18.22.

The color of the product became darker due to the high evaporation rate at the high inlet temperature which concentrated the pigments in the raw material (Quek et al., 2007). Caliskan and Dirim (2016) concluded that the L* value increased and the a* and b* values decreased with the increase of maltodextrin concentration during the process of spray drying of the sumac extracts. In the study of Jafari et al. (2017) on the spray‐drying process of pomegranate juice, authors confirmed that increasing the inlet temperature increased the brightness of the obtained powder. In contrast, the study of Quek et al. (2007) on spray‐dried watermelon powder concluded that the decrease in L* values at high inlet temperature could be due to the high sugar content in the raw materials that gets involved in the browning reactions at high temperature. At a high maltodextrin ratio, the a* values of the powder decreased as this value is closely related to the anthocyanin content of the feed.

### 3.6 Pearson correlation between contents of phenolics, anthocyanins with antioxidant activities, and color parameters of spray‐dried roselle powder

Pearson correlation coefficients between contents of phenolics, anthocyanins with antioxidant activities, and color parameters of spray‐dried roselle powder are exhibited in Table 2. It can be observed that TPC and TAC have a strong positive correlation with the antioxidant activity values of spray‐dried hibiscus powder. In particular, the correlation coefficients between TPC and DPPH free radical scavenging activity and metal ion reduction activity (FRAP and CUPRAC) were above 0.84 (*p* < .01). Our other study on the microencapsulation of roselle anthocyanins using spray‐drying and freeze‐drying techniques with the support of various carriers such as maltodextrin, gum Arabic, inulin, konjac glucomannan, and their combination showed a stronger correlation between TPC, TAC, and four antioxidant activity values with correlation coefficients all greater than 0.97 (*p* < .01). This finding led to taking into consideration the role of different carriers and operating temperatures in selectively protecting and encapsulating different bioactives (data not shown). de Souza et al. (2014) also figured out that there was a high relationship between the content of antioxidants in pomace powder and antioxidant capacity with correlation coefficients all higher than 0.98. Many polyphenolic compounds, especially those containing catechol or pyrogallol nuclei such as anthocyanins, can act as electron or proton donors, which transfer electrons to effectively reduce free radicals. The antioxidant mechanism of anthocyanins is found on this principle (Dangles & Fenger, 2018). Furthermore, the number of free hydroxyl groups in the pyrone ring that may bind with metal ions might show the secondary antioxidant mechanism of anthocyanins (Sarma et al., 1997).

**TABLE 2:** Pearson correlation coefficients between the contents of phenolics (TPC), anthocyanins (TAC) with antioxidant activities (DPPH free radical scavenging activity, ABTS cation radical scavenging capacity, ferric reducing antioxidant power—FRAP, and cupric reducing antioxidant capacity—CUPRAC) and color indicators (L*, a*, b*, C*, and h°) of spray‐dried roselle powder

	TPC	TAC	DPPH	ABTS	FRAP	CUPRAC	L*	A*	b*	C*	h°
TPC	1										
TAC	0.918**	1									
DPPH	0.843**	0.856**	1								
ABTS	0.718**	0.703**	0.875**	1							
FRAP	0.864**	0.775**	0.924**	0.812**	1						
CUPRAC	0.879**	0.884**	0.970**	0.872**	0.936**	1					
L*	−0.696**	−0.584*	−0.777**	−0.852**	−0.869**	−0.819**	1				
a*	−0.758**	−0.753**	−0.620**	−0.543*	−0.589*	−0.586*	0.429	1			
b*	0.064	−0.134	−0.189	−0.080	0.055	−0.040	−0.189	0.164	1		
C*	−0.741**	−0.0752**	−0.625**	−0.540*	−0.575*	−0.580*	0.407	0.997**	0.238	1	
h°	0.686**	0.540*	0.397	0.412	0.543*	0.473*	−0.506*	−0.722**	0.563*	−0.668**	1

*Correlation is significant at the 0.05 level (2‐tailed).

**Correlation is significant at the 0.01 level (2‐tailed).

Although there is a strong relationship between TPC and antioxidant activities, the TPC value does not account for all of the antioxidants found in hibiscus raw materials. According to Georgetti et al. (2008), during spray drying at high temperature, chemical reactions occurring between antioxidants as well as interactions with carriers can also lead to changes in antioxidant activity. As mentioned above, high temperatures may have a detrimental effect on the structure of phenolic compounds, inducing disruption and the production of different molecules, lowering antioxidant ability.

Regarding the color of spray‐dried powder, the primary coloring components responsible for the distinctive red color of roselle extracts and powder are anthocyanins. The most important values were deemed to be luminance (L*) and red (a*). In most cases, the red value rises with higher anthocyanin concentration. However, Gonçalves et al. (2007) found that when the content of this compound exceeds the "inversion area," where the L*, a*, and b* are not related in a linear way with anthocyanins, the color values appear to be inversely proportional to the anthocyanin content, resulting in the sample color darkening. The aforementioned finding was confirmed by the connection between a* value and anthocyanin content. Furthermore, the increased anthocyanin content caused the extracts and powder to become a deeper red hue, lowering the brightness and chroma of the samples. This result was also supported by Archaina et al. (2019), who discovered that as anthocyanin concentrations rose, the color attributes of spray‐dried hibiscus powder samples decreased.

## 4 CONCLUSIONS

Hibiscus powder can be obtained using the spray drying process with the assistance of maltodextrin as carrier, in which inlet temperature and carrier ratio are two important parameters affecting the quality of the product. Because maltodextrin only works as a bulking agent or a spray drying aid, increasing the amount of carrier offered greater protection of antioxidants but lowered product quality. Increased temperature in the range of 150–170°C, on the other hand, lowered anthocyanin concentration and antioxidant activities while increasing the phenolic content of the product. The temperature in the investigated range also had an insignificant effect on the color of the product while the high amount of carrier improved the brightness of the product. In addition, high solubility allows the pigment powder to be introduced into other food products, especially beverages, as colorants.

## CONFLICT OF INTEREST

The authors declared no potential conflicts of interest.

## AUTHOR CONTRIBUTIONS

**Quoc‐Duy Nguyen:** conceptualization (equal) ; data curation (equal); methodology (equal); writing original draft (equal); writing review editing (equal). **Thanh‐Thuy Dang:** conceptualization (equal) ; methodology (equal); writing review editing (equal). **Thi‐Thuy‐Dung Nguyen:** data curation (equal) ; investigation (equal). **Nhu‐Ngoc Nguyen:** investigation (equal) ; writing review editing (equal).

## Data Availability

The data that support the findings of this study are available from the corresponding author upon reasonable request.
